# Implementation and Evaluation of an AI-Assisted Telerehabilitation System for Postdischarge Continuation of Rehabilitative Care: Protocol for a Randomized Controlled Trial

**DOI:** 10.2196/78400

**Published:** 2026-08-05

**Authors:** Charmaine You Mei Tan, Xin Yi Seah, Sharna Si Ying Seah, Xin Hui Loh, Wendelynn Hui Ping Chua, Olivia Jiawen Xia, Chitra D/O Chandran, Hozaidah Hosain, Wenjing Qiu, Kevin Chong, Bee Choo Tai, Lian Leng Low

**Affiliations:** 1 Department of Post-Acute and Continuing Care Outram Community Hospital Singhealth Community Hospitals Singapore Singapore; 2 Research and Translational Innovation Office Singhealth Community Hospitals Singapore Singapore; 3 Department of Physiotherapy Sengkang Community Hospital Singhealth Community Hospitals Singapore Singapore; 4 Department of Physiotherapy Outram Community Hospital Singhealth Community Hospitals Singapore Singapore; 5 Department of Physiotherapy Jurong Community Hospital Singapore Singapore; 6 Saw Swee Hock School of Public Health National University of Singapore Singapore Singapore

**Keywords:** rehabilitation, wearable, wearable device, mobile device, exercise, exercise program, telerehabilitation, community hospital, artificial intelligence

## Abstract

**Background:**

The World Health Organization (WHO) launched a Rehabilitation 2030 initiative to call for global action to scale up rehabilitation efforts. Rehabilitation needs are growing, and efforts should be made to strengthen and integrate rehabilitation into all levels of health care, including building research capacity and expanding evidence for rehabilitation. Postdischarge rehabilitation is essential for reducing hospital readmissions and maintaining patients’ health within the community. However, locally, the shift toward community-based rehabilitation is often hampered by long waiting times for admission to day rehabilitation centers (DRCs), cost and logistical barriers, and the lack of a structured program to onboard patients and caregivers to the digital solutions required for rehabilitation in Singapore. Telerehabilitation systems that incorporate wearables within a structured rehabilitation program support inpatient rehabilitation and enable patients to continue with physical rehabilitation after discharge while awaiting admission to a DRC.

**Objective:**

The aim of this randomized controlled trial (RCT) is to investigate the clinical and cost-effectiveness of ATLAS, an AI-assisted telerehabilitation system, among patients admitted to community hospitals (CHs) for rehabilitation.

**Methods:**

This is a 2-arm pragmatic RCT. Participants admitted to CHs for rehabilitation for hip fracture, musculoskeletal conditions, or deconditioning will be enrolled and randomized to either the intervention or control group in a 1:1 ratio. The intervention group, in addition to usual care, will be enrolled in an ATLAS system. The ATLAS system consists of Rebee, an AI-assisted device equipped with a lightweight wireless motion sensor that patients can strap on for real-time feedback and log their exercises via the Rebee application to an electronic platform, as well as a therapist-designed, structured exercise program that patients follow during their inpatient stay and continue upon discharge. The control group will continue to receive usual rehabilitative care in the CHs and upon discharge but will not have access to the ATLAS system. Our primary outcome is functional status. Secondary outcomes include quality of life, length of stay in the CH, 30-day readmission rates, and cost-effectiveness.

**Results:**

A total of 407 participants were recruited between January and September 2025 across 3 CHs, with data collection completed in December 2025. Data analysis is currently underway, and the results are expected to be submitted for publication in Q4 2026.

**Conclusions:**

Our trial will provide valuable insights into the effectiveness and implementation of an AI-assisted telerehabilitation system for patients admitted to the CH for rehabilitation for hip fracture, musculoskeletal conditions, or deconditioning. This trial will also evaluate the sustainability and cost-effectiveness of the ATLAS system, with the potential of scaling across inpatient settings in both acute and CHs.

**Trial Registration:**

ClinicalTrials.gov NCT06683963; https://clinicaltrials.gov/study/NCT06683963

**International Registered Report Identifier (IRRID):**

DERR1-10.2196/78400

## Introduction

The world’s population is aging, and by 2030, 1 in 6 people will be aged ≥60 years [1]. The rapidly ageing population has greatly contributed to the present and future increase in health care costs, with an increase in long-term care expenditures for older individuals [2]. Similarly, in Singapore, the proportion of older adults aged ≥65 years increased from 11.2% in 2014 to 18% in 2024 [3]. In 2017, the World Health Organization (WHO) launched a Rehabilitation 2030 initiative to call for global action to scale up rehabilitation efforts [4]. This initiative recognizes that rehabilitation needs are increasing, and efforts should be made to strengthen and integrate rehabilitation into all levels of health care. This includes building research capacity and expanding evidence for rehabilitation.

Studies have shown that early postdischarge rehabilitation is essential for preventing deterioration, reducing readmissions, and maintaining patients’ health within the community [5-8]. Locally, there have been efforts to improve patients’ access to an expanded scope of community rehabilitation by right-siting suitable patients from acute hospitals and specialist outpatient clinics to community-based rehabilitation facilities, such as day rehabilitation centers (DRCs) [9]. However, the waiting time for DRCs in Singapore can range widely due to limited capacity, alongside other factors, resulting in a median waiting time for day centers of around 20 days [10]. As such, solutions are required that allow for early access to postdischarge rehabilitation, apart from that which is provided by the community rehabilitation facilities.

Besides the availability of community rehabilitation facilities, adherence to postdischarge rehabilitation at these facilities has also been an issue, with postdischarge rehabilitation attendance rates dropping steadily over time [8,11]. The number of rehabilitation sessions attended is closely related to functional outcomes [12]. Barriers to adherence include (1) functional limitations and inconvenience in traveling to rehabilitation centers, (2) caregiver availability, and (3) financial reasons [11,13]. As such, research and development of telerehabilitation has been rapidly expanding to address the above barriers, demonstrating that digital health (DH) care solutions may even perform better than conventional rehabilitation in reducing pain and improving quality of life [14]. Published trials on telerehabilitation have used a combination of methods, ranging from digital devices to home visits and video- or phone-based interventions [15]. However, home-based therapy and traditional telerehabilitation require manpower for visitation and consultation, resulting in additional manpower costs.

The shift toward community-based rehabilitation thus requires a digital solution that should be convenient and most importantly AI-assisted to reduce manpower costs. Studies evaluating unsupervised home exercises via a digital platform or printed programs have been shown to be noninferior to formal outpatient supervised therapy sessions [16-18]. The digital literacy of Singapore’s older adults varies greatly; thus, help is required to smoothen the process of DH adoption and uptake. This will range from providing education on the DH interface and navigation, to ensuring ease of access and support to use of DH devices, recommendations by trusted health care institutions and health care professionals, convenience, provision of free DH devices or monetary incentivization, as well as aligning with participants’ own healthy living goals [19,20].

The Singapore Tele-technology Aided Rehabilitation in Stroke study, which was a randomized controlled trial (RCT) conducted on 124 patients who had a stroke in Singapore, compared a 12-week telerehabilitation system and program to usual care for recent stroke survivors [21,22]. The study demonstrated the feasibility of such self-directed home rehabilitation devices, with similar improvements in functional outcomes noted between both groups. To our knowledge, apart from the above-mentioned study, there are no other clinical trials examining the feasibility, outcomes, and most importantly cost-effectiveness of implementing such self-directed telerehabilitation programs in any other groups of patients in Singapore.

To address this, the team designed the ATLAS study, a 2-arm (intervention-control) RCT conducted across 3 community hospitals (CHs) in Singapore, to evaluate the outcomes and cost-effectiveness of the addition of telerehabilitation systems to usual care, for patients admitted to CHs for rehabilitation for hip fracture, musculoskeletal conditions, or deconditioning. Rebee is a Health Sciences Authority class A certified medical device that has been validated in previous studies locally and abroad for measurement of upper and lower limb range of motion and is approved for clinical trials across the 3 health care clusters in Singapore [23-25]. Rebee consists of a tablet with an attached lightweight wireless motion sensor that patients can strap on for real-time feedback and log their exercises via the Rebee application to an electronic platform that can be viewed by the study team.

Singapore CHs provide postacute care environments characterized by lower levels of anxiety, extended patient stays for rehabilitation, and opportunities for caregiver training [26]. CHs are ideal environments for trials using DH devices, given that patients stay inpatient for up to several weeks for rehabilitation, allowing time for dedicated hospital personnel to familiarize, introduce, and teach patients and their caregivers in the intervention arm how to use DH devices during their stay.

The primary aim of this RCT is to determine if the addition of ATLAS to usual care improves functional status, as compared to usual care alone, in patients admitted to CHs for rehabilitation for hip fracture, musculoskeletal conditions, or deconditioning. The secondary aim is to determine if this also affects patients’ quality of life, length of stay in CHs, 30-day readmission rates, and clinical cost-effectiveness, as compared to usual care alone. The hypothesis is that the addition of ATLAS to usual care improves functional status and quality of life, reduces length of stay in CHs and 30-day readmission rates, and increases clinical cost-effectiveness, as compared to usual care alone. The findings from this study may provide evidence for an enhanced, cost-effective, and sustainable care model for patients admitted for rehabilitation to a CH.

## Methods

### Study Design

**Figure 1:**
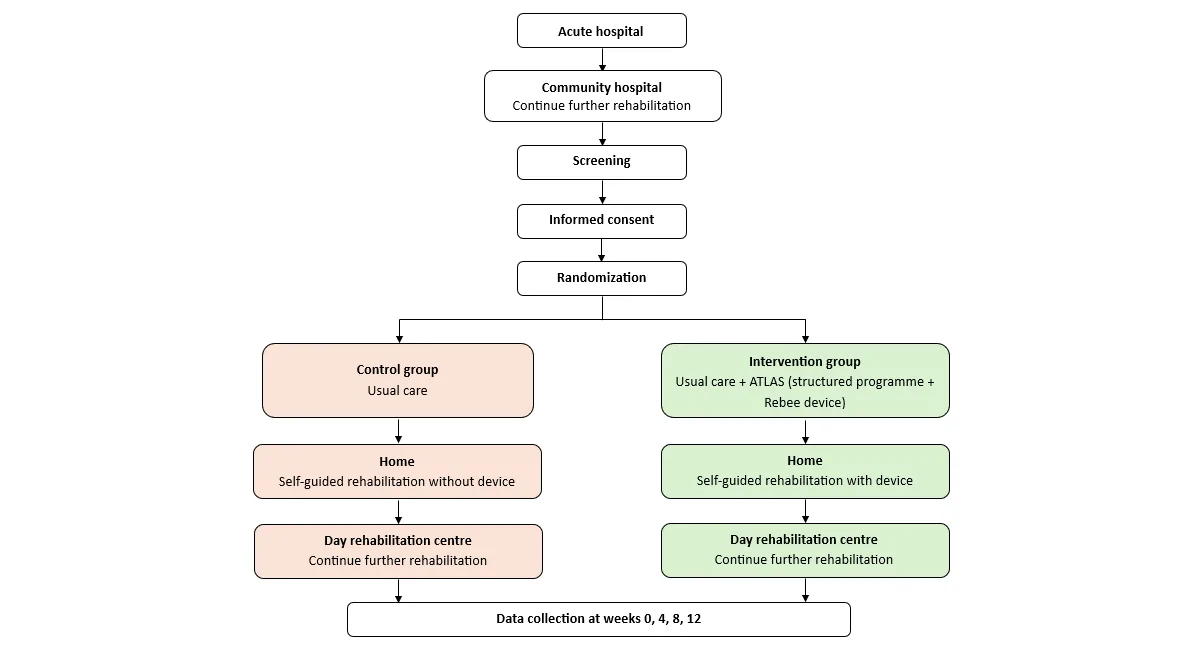
ATLAS trial workflow.

**Figure 2:**
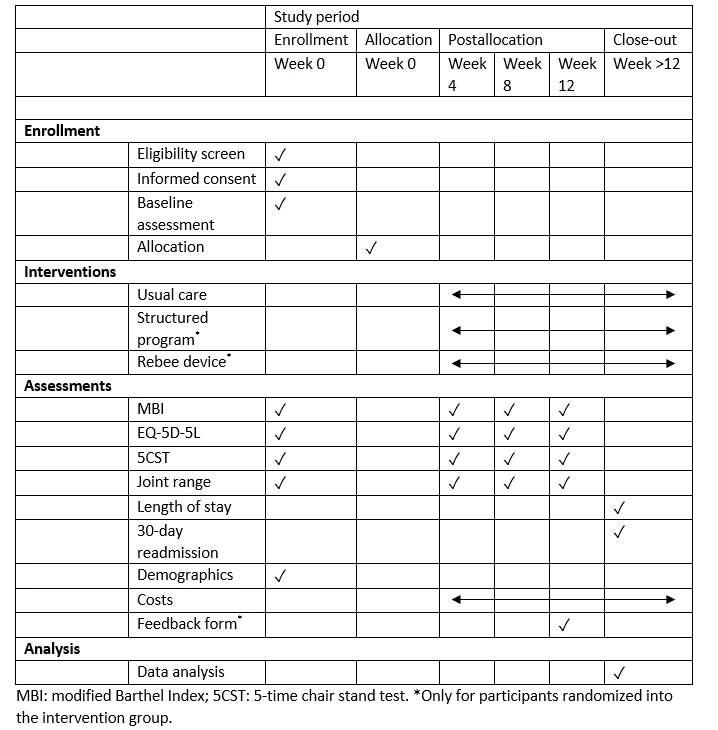
SPIRIT (Standard Protocol Items: Recommendations for Interventional Trials) checklist for the schedule of enrollment, interventions, and assessments.

This study is an unblinded, multicenter, prospective, pragmatic parallel 2-arm RCT comparing the efficacy and cost-effectiveness of a 12-week application-based telerehabilitation program to usual care across 3 CHs in Singapore. Patients recruited at each site will be randomly allocated in a 1:1 ratio to either an intervention or control group. The intervention group will be enrolled into ATLAS, consisting of a structured exercise program and Rebee, an AI-assisted telerehabilitation device, in addition to usual care. The control group will continue to receive usual care but will not have access to ATLAS (Figure 1). The study design is based on the Pragmatic Explanatory Continuum Indicator Summary Framework-2 (PRECIS-2) [27]. This study protocol is written according to the Standard Protocol Items: Recommendations for Interventional Trials reporting guidelines [28] (Figure 2). Study outcomes will be reported in accordance with the Consolidated Standards of Reporting Trials statement [28].

### Study Setting

This study was conducted in 3 CHs (Outram Community Hospital, Seng Kang Community Hospital, and Jurong Community Hospital) in Singapore. CHs are purpose-built hospitals that provide medical, nursing, and rehabilitation care for patients who require a short period of care after their stay in the acute hospital before being discharged back to the community [26]. Patients admitted to CHs may come from any acute tertiary hospital in Singapore.

### Ethical Considerations

This study has been approved by the SingHealth Centralised Institutional Review Board (2024-2246) and is registered on ClinicalTrials.gov (NCT06683963). Written informed consent was obtained from all participants before study participation. Participants on each site are assigned a continuous study code to protect identifiable data (eg, OCH001-OCH150, SKCH001-SKCH150, and JCH001-JCH150). The assignment data are kept in a password-protected laptop, within a password-protected document, and only accessible by selected study team members. Participants are compensated for their time and inconvenience via grocery vouchers given upon completion of predefined time points in the study (Singapore $20 [Singapore $1=US $0.77 as of July 21, 2026] before discharge and Singapore $30 at the end of the study).

To ensure confidentiality of participants, only the study team will have access to the research data, both hard and soft copies, at all sites. Rebee Health will only receive deidentified and nonsensitive data. Hard copy data will be stored in designated locked locations (eg, cabinets and rooms) that are accessible to only authorized study personnel. Electronic data will be stored in an institution-approved, secured, and encrypted storage medium, such as databases, encrypted portable media (eg, USB drives, CD or DVD, and hard disks), and/or institution-approved online storage platforms. The electronic data will not contain research participant identifiers. Identification codes linking electronic data and research participants will be stored separately. The research data will be destroyed after they have been stored for the minimum duration of retention as specified by the institutional policy.

### Eligibility Criteria

Patients will be recruited from 3 CHs in Singapore. The inclusion criteria are as follows: (1) age between 21 and 99 years; (2) admission primary rehabilitation diagnostic group (RDG) classified under hip fracture (3.11, 3.12, and 3.13), musculoskeletal conditions (5.11, 5.12, 5.13, and 5.21), or deconditioning (6.1, 6.2, and 6.3) [9]; (3) understand English or Mandarin; and (4) suitable and able to engage in Rebee exercises.

The exclusion criteria are as follows: (1) colonized by multi-drug-resistant organism; (2) presence of neuropsychiatric or neurocognitive disease affecting the ability to follow instructions; (3) unable to full weight bear, or restrictions to range of motion; and (4) refusal to participate or give informed consent.

### Usual Care

Patients admitted to CHs undergo inpatient rehabilitation for around 3 to 4 weeks before being discharged home. During the CH admission, participants in both arms will receive standardized CH rehabilitation comprising both physiotherapy and occupational therapy sessions. Physiotherapy and occupational therapy sessions are each held for 30 to 60 minutes per session, up to 5 times a week, covering a range of items including warm-up, strength training, cardiovascular training, and balance training. All training may be conducted in either a group or one-to-one setting based on clinical needs and patient safety considerations [26]. Caregiver training is provided to patients and their respective family members or caregivers as needed prior to discharge.

All therapists delivering rehabilitation services are registered under the Allied Health Professions Council, a professional board that is governed and regulated by the Ministry of Health. This ensures standardization of professional qualifications across both study arms.

After discharge from a CH, participants will be referred to outpatient rehabilitation facilities as recommended by the CH team (eg, center-based therapy at DRCs or outpatient therapy clinic, or home-based therapy) as per usual care. They will be prescribed exercises communicated via printed materials, verbal instruction, and demonstration.

### Intervention

During their CH stay, patients in the intervention arm will be enrolled in the ATLAS program in addition to usual care (Figure 1). The ATLAS program is provided free of charge to the patients and their caregivers, with follow-up and support provided during their stay as well as upon discharge for up to 12 weeks. It consists of Rebee, an AI-assisted telerehabilitation device with a wearable sensor, and a structured rehabilitation program.

#### Rebee

**Figure 3:**
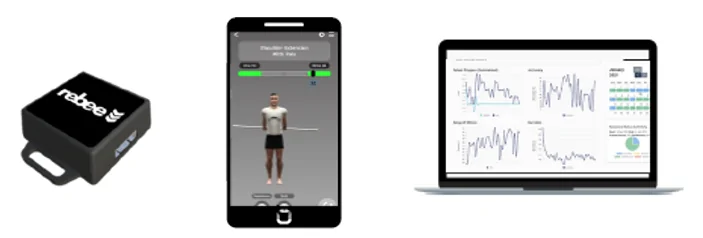
Wearable sensor, Rebee application, and therapist web portal. More information can be found on the Rebee Health website.

**Figure 4:**
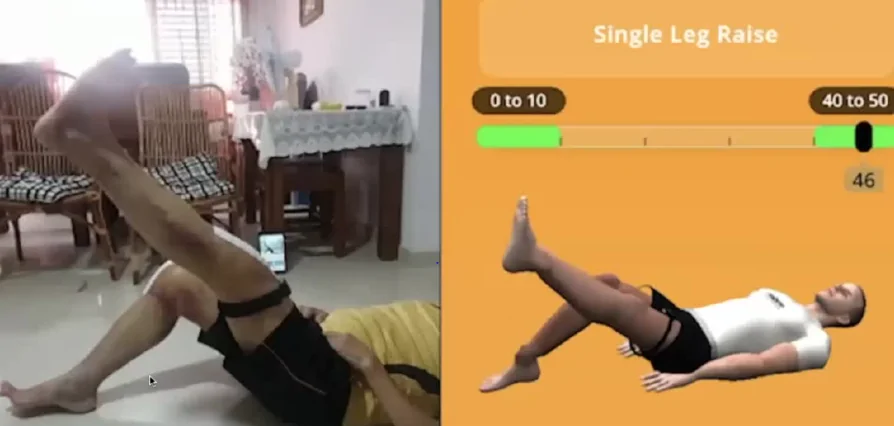
Rebee virtual 3D avatar with real-time tracking and feedback of movements and range.

Participants in the intervention arm will be provided a tablet containing the Rebee application and a linked wearable sensor (Figure 3). The Rebee interface is simple and intuitive to use, with video demonstrations on how to perform the exercises and a virtual 3D avatar that translates movements detected by the linked sensor into visual and audio cues to provide real-time feedback and gamification of exercises (Figure 4).

As Rebee’s clinical platform has been whitelisted in SingHealth and National University Health System, study team members will be able to access the Rebee therapist web portal from their corporate devices to access and monitor participants’ progress.

#### Structured Program

Upon recruitment, the study team will upload a set of inpatient rehabilitation exercises to the Rebee portal, with the type of exercise, number of sets, and number of repetitions predetermined according to the participant’s RDG grouping (Tables 1 and 2). These exercises were decided upon via consensus by the lead therapists in the study team across all 3 CH sites, modeled after deemed safe for patients to perform on their own without supervision from the inpatient rehabilitation team. The study team will then visit the participant daily to provide guidance and training on the use of the Rebee device, until the participant and/or their caregivers are deemed competent to use the device on their own. Participants will be provided phone numbers to contact the study team if they have issues or questions.

**Table 1:** List of inpatient exercises.

TKR^a^	All others^b^
Isometric quads (ISO knee extension; 2 sets × 5 reps, ISO 5 s hold)	Bridging (2 sets × 5 reps)
Single-leg raise with towel (2 sets × 5 reps, ISO 5 s hold)	Supine heel slide (left and right; 2 sets × 5 reps)
Passive knee flexion with towel (2 sets × 5 reps, ISO 5 s hold)	Supine hip abduction (left and right; 2 sets × 5 reps)
Seated knee extension (2 sets × 5 reps, ISO 5 s hold)	Single-leg raise (left and right; 2 sets × 5 reps)
Seated marching (3 sets × 1 min, 30 s rest in between)	Seated knee extension (left and right; 2 sets × 5 reps, ISO 5 s hold)

^a^TKR: total knee replacement.

^b^This includes all participants under rehabilitation diagnostic group hip fracture (3.11, 3.12, and 3.13), musculoskeletal conditions (5.11, 5.12, and 5.21), and deconditioning (6.1, 6.2, and 6.3), except for total knee replacement (5.13).

**Table 2:** List of outpatient exercises.

TKR^a^	All others^a^
Isometric quads (ISO knee extension; 3 sets × 10 reps, ISO 5 s hold)	Bridging (2 sets × 10 reps)
Single-leg raise with towel (3 sets × 10 reps, ISO 5 s hold)	Supine heel slide (left and right; 2 sets × 10 reps)
Passive knee flexion with towel (3 sets × 10 reps, ISO 5 s hold)	Supine hip abduction (left and right; 2 sets × 10 reps)
Seated knee extension (3 sets × 10 reps, ISO 5 s hold)	Single-leg raise (left and right; 2 sets × 10 reps)
Brisk marching on the spot (3 sets × 1 min, 30 s rest in between)	Seated knee extension (left and right; 2 sets × 10 reps, ISO 5 s hold)

^a^TKR: total knee replacement.

^b^This includes all participants under rehabilitation diagnostic group hip fracture (3.11, 3.12, and 3.13), musculoskeletal conditions (5.11, 5.12, and 5.21), and deconditioning (6.1, 6.2, and 6.3), except for total knee replacement (5.13).

Participants are to follow and perform the preset exercises on the Rebee device in their own time, in addition to routine rehabilitation sessions conducted during their stay in the CHs. Upon discharge, the preset exercises will be updated to an outpatient regime (Table 2), and participants will be advised to continue performing these exercises on their own.

Study team members will check in with the participants if the Rebee device has not been used for 3 or more consecutive days to troubleshoot or understand reasons for lack of use. Participants are allowed to discontinue the Rebee device upon request, and reasons for such discontinuation are recorded (eg, not interested anymore and unable to continue using the Rebee device due to reasons such as injury, admission to hospital, or travel). The Rebee device will be retrieved at the end of the study at week 12 through a scheduled home visit.

### Outcome Measures

The primary outcome is functional status measured over 12 weeks. Functional status will be measured using modified Barthel index (MBI), a 100-point ordinal scale for the assessment of a patient’s functional state. The MBI assesses activities of daily living over 10 domains comprising transfers, ambulation, stair climbing, bathing, dressing, toileting, bowel control, bladder control, personal hygiene, and feeding [29].

Secondary outcomes include physical performance, quality of life, length of stay in CH, 30-day readmission rates, and cost-effectiveness over 12 weeks. Patients who are admitted under RDG total knee replacement will have an additional outcome measure of joint range. The outcome measures as well as the method of measurement are listed in Table 3. Health care cost will be calculated from electronic medical records and supplemented with survey data regarding the number of DRC or home therapy sessions attended, manpower hours to train patients to use Rebee, costs of Rebee software and hardware, and use of outpatient community services (eg, day care center, home nursing, housekeeping, and meals on wheels). The tools to be used in this study to measure the outcomes are validated and implemented in routine clinical care.

**Table 3:** Primary and secondary outcome measures.

Variables			Data source, method of measurement, and definitions		Time points
Primary outcome					
	Functional status	Survey: MBI^a^. To establish the degree of independence from any help. A score from 0- 100 using the MBI questionnaire, with 100 being fully ambulant without any help.		Baseline and 4, 8, and 12 weeks	
Secondary outcomes					
	Joint range	Measurement using goniometer on affected knee. Active and passive range of motion (flexion and extension) of affected knee.		Baseline and 4, 8, and 12 weeks	
	Physical performance	Measured by performing 5CST^b^. Measures the time taken to complete 5 sit to stands (in seconds)		Baseline and 4, 8, and 12 weeks	
	Quality of Life	Survey: EQ-5D-5L		Baseline and 4, 8, and 12 weeks	
	Length of stay	Electronic medical records		Date of discharge from CH^c^ minus date of randomization	
	30-day readmission rates	Electronic medical records		12 weeks	
	Direct health care cost	Electronic medical records: cost of inpatient stay		12 weeks	
	Direct health care cost	CRC’s^d^ daily expenditure of manpower		4, 8, and 12 weeks	
	Direct health care cost	Rebee costs: software and hardware		12 weeks	

^a^MBI: modified Barthel index.

^b^5CST: 5-time chair stand test.

^c^CH: community hospital.

^d^CRC: clinical research coordinator.

### Sample Size

We calculated the sample size based on the primary outcome of MBI scores, which is evaluated at recruitment and at 4, 8, and 12 weeks. The historical mean of the MBI is 73 (SD 25). The study aims to detect a difference of 5 points between the control and intervention groups assuming a correlation of 0.35 between the repeated measurements. The sample size estimated to detect this difference with 80% power and a 5% significance level is 175 participants per group assuming equal allocation between arms. After accounting for 20% attrition, the total sample size required for the study is 450.

### Recruitment and Screening

Participants will be recruited from the inpatient wards of 3 CHs, namely Outram Community Hospital, Sengkang Community Hospital, and Jurong Community Hospital. Each site will recruit 150 patients (75 in the intervention group and 75 in the control group), with a total of 450 participants recruited across all 3 sites.

The participants will first be screened by their ward therapists for eligibility, after which a member of the research team will approach them to provide information about the study as well as to take informed consent. Study team members will also provide reminders to ward therapists during their regular roll calls to maximize recruitment efforts.

### Randomization and Blinding

Participants will be randomized to the intervention or control group based on a 1:1 allocation ratio. Stratified block randomization with randomly varying block sizes will be implemented with recruitment site as the stratification factor.

After taking informed consent, the research coordinator will contact a member of the team (who oversees the progress but is not involved in patient recruitment) to obtain the randomized assignment. The research coordinator will remain blinded to the allocation prior to contacting the team member. Due to the nature of the intervention, blinding of study participants will not be possible.

### Statistical Analyses

Participants will be analyzed via an intention-to-treat approach. Participant characteristics will be summarized using median (IQR) or mean (SD) for nonnormally distributed and normally distributed data, respectively, and count and percentage for categorical variables.

Linear mixed-effects model will be used to assess the pattern of change in the primary outcome of the MBI score and secondary outcomes of EQ-5D, 5-time chair stand test (5CST), and joint range over the 12-week follow-up period. A uniform correlation structure will be specified to model the within-subject correlation of these repeated measures. MBI, EQ-5D, 5CST, and joint range of motion will be treated as continuous outcomes. Normality of continuous outcome variables will be assessed through visual inspection methods. If normality assumptions are met, parametric methods will be used; otherwise, a suitable transformation may be performed to address the issue of nonnormality.

As individuals of different age groups have varying body composition, functional capacity, and physiological reserves, there may be differential influences on baseline functional ability and recovery trajectories. To ensure that any potential age-related differences are accounted for, we will first assess the interaction between the age category, treatment groups, and duration of time. We will stratify into age categories, and if statistically meaningful effect modification by age is observed, an age-stratified analysis will then be conducted.

Poisson regression will be used to compare length of stay between intervention and control groups. If there is evidence of overdispersion and/or zero inflation, negative binomial regression or corresponding zero-inflated models will be implemented instead.

Key prognostic factors such as baseline MBI, RDG grouping, and time will be adjusted a priori. The secondary outcome based on 30-day readmission will be compared between the intervention and control group using modified Poisson regression. The effect estimate will be quantified in terms of relative risk and its 95% CI.

For both the primary and secondary outcomes, linear mixed-effects model on the log-transformed scale will be applied if the model assumptions are violated.

Missing data are expected due to the nature of the study and will be handled by imputation.

The preferred method of imputation will be applied accordingly based on the degree of missingness, such as last observation carried forward.

The cost-effective analysis will evaluate both direct cost savings and cost-effectiveness in terms of quality-adjusted life years (QALYs). To assess direct cost savings, the analysis will evaluate the difference in length of stay at the hospital between the intervention and control group (Table 4). The results will be expressed as mean cost differences between groups. Cost per QALY gained will be evaluated for the cost-effectiveness of the intervention. QALY will be derived using EQ-5D-5L utility scores, and incremental cost-effectiveness ratios (ICERs) will be calculated. The ICERs will be compared to established willingness-to-pay thresholds to determine the cost-effectiveness of the intervention. Sensitivity analyses will be performed to account for uncertainties in cost and QALY estimates. Results will be presented using cost-effectiveness planes and acceptability curve.

**Table 4:** Cost-accounting for the cost-effectiveness analysisa.

Cost category	Cost type	Cost item	Unit of measurement	Source of unit cost	Applicable to
Hardware	Direct	Rebee kits	Per kit per day	Research collaboration agreement	Intervention
Software	Direct	Rebee subscription	Per patient per day	Research collaboration agreement	Intervention
Inpatient care	Direct	Average length of stay	Per patient per day	Electronic medical records	Control and intervention
Manpower	Direct	CRC’s^b^ daily EOM^c^	Per day	Human resource	Intervention

^a^The statistical analysis will be conducted using R (R Foundation for Statistical Computing). All evaluations will be made using 2-side *P* values at the 5% level of significance.

^b^CRC: clinical research coordinator.

^c^EOM: expenditure of manpower.

### Implementation

Feedback on the Rebee device and application will be collected from the intervention group through 6 close-ended questions and 1 open-ended question at the end of the study (Multimedia Appendix 1). Specifically, feedback will be collected on the ease of use of the Rebee app and exercises, the ability to complete Rebee exercises independently, any technical issues encountered, the overall user experience, the suggested improvements, and whether they would recommend this technology to others. A simple descriptive analysis will be conducted to summarize the quantitative findings using frequencies and percentages. A content analysis will be performed on the qualitative responses to identify keywords and phrases. If there is substantial variation, we will conduct a thematic analysis to summarize the common themes.

### Adherence Monitoring

Rebee use data for each participant will be retrieved and analyzed via the Rebee software [25]. Use data will be broken down in detail to evaluate daily compliance rate to use of Rebee and its exercises, as well as overall use and uptake over the 12-week period.

### Data Management and Monitoring

#### Overview

All hard copy data will be stored in locked storage spaces at the respective recruitment sites during the study. At the end of the study, all hard copy data will be stored together at one of the recruitment sites. Only authorized study personnel will be able to access these locked cabinets. Survey data will be entered into a password-protected Excel worksheet. We will retrieve data from the electronic medical records upon participant consent. All softcopy data will be password-protected and only accessible to the study team. There is no identifiable data collected on the Rebee application, and only the Rebee team will have access to the nonsensitive data (eg, frequency and duration of rehabilitation exercises).

At the end of the study, all data stored in the Rebee application will be deleted. All softcopy and hard copy data will be maintained for 7 years after study termination and then disposed of in accordance with policy.

Given the minimal risk presented in the use of the wearable device, this study does not involve a data monitoring committee. However, the study team will continuously monitor participant safety through regular reviews of any reported adverse events (AEs), and if any safety concerns arise, the team will report them to the ethics board promptly following the required institutional procedures. There is no interim analysis planned for this study.

#### Trial Monitoring

Safety monitoring will involve monthly checks for any AEs and serious AEs at scheduled time points and whenever needed. The study team will carry out periodic data quality reviews and conduct random monitoring visits to ensure the study is being followed correctly and that data collected is accurate and complete.

## Results

**Figure 5:**
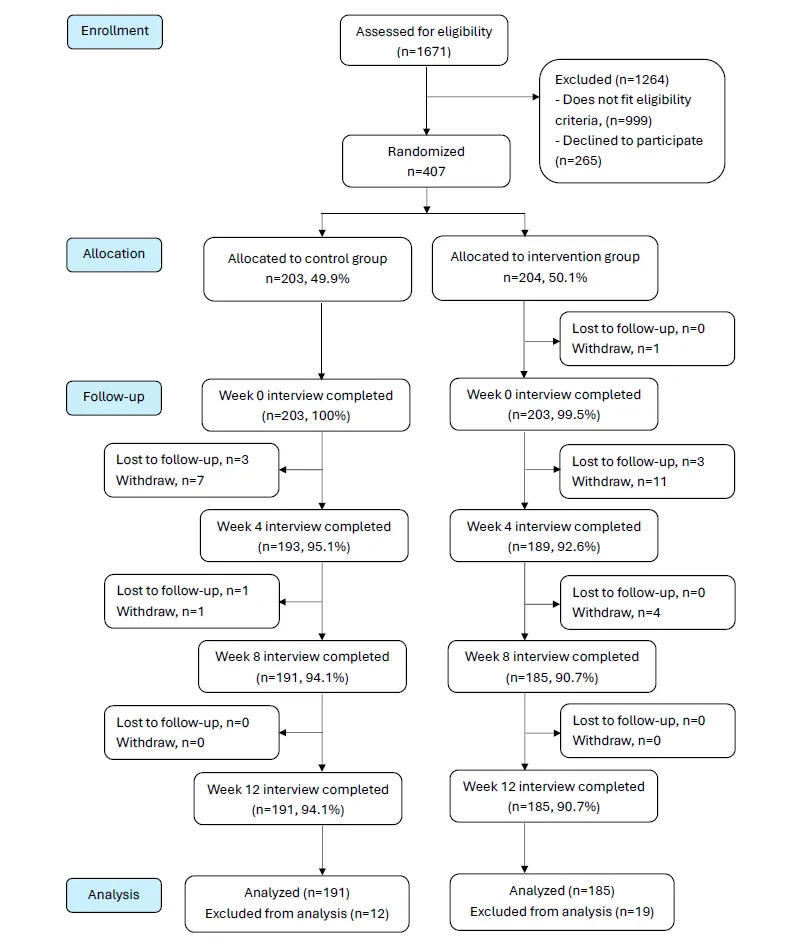
Data collection and analysis workflow.

Recruitment took place from January 6, 2025, to September 23, 2025. A total of 407 participants were recruited from January to September 2025, with data collection completed in December 2025. Figure 5 shows the participant progression during the conduct of our study. Data analysis is currently underway, and the first results are expected to be submitted for publication in Q4 2026.

## Discussion

### Anticipated Findings and Comparison to Prior Work

To the best of our knowledge, this study is the first to determine the clinical effectiveness and cost-effectiveness of an AI-assisted telerehabilitation device with a structured training program starting from inpatient stay and continued upon discharge via an RCT study design among patients requiring postdischarge rehabilitation care in Singapore. We anticipate that patients receiving the telerehabilitation device and structured training program will demonstrate greater improvement in their functional status, as measured by the MBI, compared to those receiving standard rehabilitation alone. We also expect improvements in joint range, physical performance, and quality of life, with reduction in length of CH stay, lower 30-day readmission rates, and increased cost-effectiveness as compared to usual care alone.

Previous studies have demonstrated that telerehabilitation devices can achieve comparable outcomes compared to conventional rehabilitation services [14,15,22]. However, studies are heterogeneous in terms of the type of intervention and technology used, with varying combinations of in-person or digital personnel required for supervision and execution of the program.

### Strengths and Limitations

This trial will contribute in several ways to existing knowledge and research regarding telerehabilitation devices and rehabilitation programs. First, it will test user acceptability and compliance with telerehabilitation devices and systems to guide the practicability of application of such devices to routine clinical practice. Second, this will evaluate the cost-effectiveness of such systems versus usual care alone to provide an economic evaluation encompassing resource use, patient-reported quality of life, and physical function improvement. Finally, this will also test whether the addition of such a telerehabilitation system as compared to usual care alone will improve outcomes in terms of functional improvement, length of stay in a CH, and 30-day readmission rates.

There are some limitations to this study. First, it was not possible to blind participants as the intervention involves receiving a physical Rebee device as well as physical sessions to teach the participant about the use of the Rebee device. This might change the behavior and response of participants in the intervention group, causing them to provide more positive outcomes. The difficulty in blinding participants may also increase patient dropout in the control arm as they do not perceive any immediate benefits to them being in the study. Second, the total health care costs calculated in the cost-effectiveness analysis may be underestimated due to omission of certain costings such as postdischarge service use costs and unplanned readmission costs. These costs were excluded because the data on postdischarge service use were collected through patient self-report and could not be verified. On the other hand, there is insufficient information on readmission costs. To minimize inaccuracy, it was decided that these costings will be excluded from the cost-effectiveness analysis. Another limitation is the language barrier, as the Rebee application is only available in English and Mandarin. Considering that Singapore is a multiracial country, this may deter participants who can only read Malay or Tamil from participating in this study. Finally, there is also a potential confounder, which is the digital literacy of participants, which was not assessed in this study. To address this issue, the study team will provide support to participants and caregivers, ensuring that they are able to operate Rebee independently.

### Dissemination Plan

Results from our study will be disseminated through peer-reviewed journals, including publications on the trial protocol, main outcomes, and secondary analyses. Results will also be presented at local and international conferences centered on rehabilitation, DH, and implementation science. Relevant findings will be provided to participating clinical sites and collaborating institutions to inform decisions regarding future adoption and scalability of such telerehabilitation technologies. Dissemination will adhere to ethical, regulatory, and data-sharing requirements.

### Future Directions

Populations around the world are rapidly aging, and health systems are attempting to bend the cost curve by shifting care from hospital-based acute care to community-based preventive health and rehabilitation. The insights on clinical and cost-effectiveness from this study shall also assist policymakers to make better decisions regarding the use of AI-assisted telerehabilitation systems to plug the present gaps in this care transition.

By incorporating both objective clinical measurements as well as health use and cost-effectiveness analyses, this trial aims to extend prior work and provide more evidence and guidance for future scaling of such telerehabilitation models.

## Data Availability

The datasets generated during and/or analyzed during this study will be available from the corresponding author upon reasonable request.

## Appendix

Multimedia Appendix 1 Feedback survey.

Multimedia Appendix 2 SPIRIT 2025 checklist.

## Abbreviations

5CST: 5-time chair stand test
AE: adverse event
CH: community hospital
DH: digital health
DRC: day rehabilitation center
ICER: incremental cost-effectiveness ratio
MBI: modified Barthel index
PRECIS-2: Pragmatic Explanatory Continuum Indicator Summary Framework-2
QALY: quality-adjusted life year
RCT: randomized controlled trial
WHO: World Health Organization
