# Supplementary material for: Implementation and Evaluation of an AI-Assisted Telerehabilitation System for Postdischarge Continuation of Rehabilitative Care: Protocol for a Randomized Controlled Trial
Source: JMIR Res Protoc. 2026 Aug 5;15:e78400. doi: 10.2196/78400 (PMC13490933; doi:10.2196/78400)
Supplement: Supplementary file 2 [file resprot_v15i1e78400_app1.docx]

**Participant code number: ______________________________**

**Visit no.:** Week 0 / Week 4 / Week 8 / Week 12

**Visit date (dd/mmm/yyyy): ____________________________**

**Feedback form for intervention group (Week 12)**

| 1. | **Daily exercises are easier to follow and complete with the Rebee Kit.** | Yes | Quite  easy | Not so  easy | Slightly  difficult | Very  difficult |
| --- | --- | --- | --- | --- | --- | --- |
| 2. | **Using the Rebee App was easy.** | Yes | Quite  easy | Not so  easy | Slightly  difficult | Very  difficult |
| 3. | **I received enough technical support (or there were no technical issues)** | Yes | Enough  support | Some  support | Not  enough support | No support  when  needed |
| 4. | **I can complete the Rebee exercises by myself** | Yes | Need  slight  help | Some  help  needed | Frequent help needed | I can’t do  them  by myself |
| 5. | **I will recommend using this technology to others.** | Yes |  | Maybe |  | No |
| 6. | **From 0 – 100, how would you rate your overall experience with Rebee?** | | | | | |
|  |  |  |  |  |  |  |
| 7. | **Any improvements that you would like to suggest on the Rebee Kit?** | | | | | |
|  | | | | | | |
|  | | | | | | |
|  | | | | | | |
|  | | | | | | |
|  | | | | | | |
|  | | | | | | |
|  | | | | | | |
|  | | | | | | |
|  | | | | | | |
|  | | | | | | |
